# Does the combination of photobiomodulation therapy (PBMT) and static magnetic fields (sMF) potentiate the effects of aerobic endurance training and decrease the loss of performance during detraining? A randomised, triple-blinded, placebo-controlled trial

**DOI:** 10.1186/s13102-020-00171-2

**Published:** 2020-04-10

**Authors:** Paulo Roberto Vicente de Paiva, Heliodora Leão Casalechi, Shaiane Silva Tomazoni, Caroline dos Santos Monteiro Machado, Neide Firmo Ribeiro, Amanda Lima Pereira, Marcelo Ferreira Duarte de Oliveira, Marjury Nunes da Silva Alves, Maiara Conceição dos Santos, Inti Ernesto Torrico Takara, Eduardo Foschini Miranda, Paulo de Tarso Camillo de Carvalho, Ernesto Cesar Pinto Leal-Junior

**Affiliations:** 1 Laboratory of Phototherapy and Innovative Technologies in Health (LaPIT), Nove de Julho University, Rua Vergueiro, 235/249, São Paulo, SP 01504-001 Brazil; 2 Postgraduate Program in Rehabilitation Sciences, Nove de Julho University, São Paulo, SP Brazil; 3 Physiotherapy Research Group, Department of Global Public Health and Primary Care, University of Bergen, Bergen, Norway; 4 ELJ Consultancy, Scientific Consultants, São Paulo, SP Brazil

**Keywords:** Low-level laser therapy, Light-emitting diode therapy, Phototherapy, Endurance exercise, Deconditioning

## Abstract

**Background:**

Photobiomodulation (PBMT) is a therapy that uses non-ionising forms of light, including low-level lasers and light-emitting diodes (LEDs) that may be capable of modulating cellular activity. Some biological processes may also interact with static magnetic fields (sMF), leading to modulatory effects on cells. Previous studies have verified that the combination of PBMT and sMF (PBMT/sMF) enhances the performance of individuals during aerobic training programs. The detraining period can cause losses in aerobic capacity. However, there is no evidence of the existence of any recourse that can decrease the effects of detraining. We aimed to investigate the effects of PBMT/sMF application during training and detraining to assess the effectiveness of this treatment in reducing the effects of detraining.

**Methods:**

Sixty male volunteers were randomly allocated into four groups— participants who received PBMT/sMF during the training and detraining (PBMT/sMF + PBMT/sMF); participants who received PBMT/sMF during the training and a placebo in the detraining (PBMT/sMF + Placebo); participants who received a placebo during the training and PBMT/sMF in the detraining (Placebo+PBMT/sMF); and participants who received a placebo during the training and detraining (Placebo+Placebo). Participants performed treadmill training over 12 weeks (3 sessions/week), followed by 4 weeks of detraining. PBMT/sMF was applied using a 12-diode emitter (four 905 nm super-pulsed lasers, four 875 nm light-emitting diodes (LEDs), four 640 nm LEDs, and a 35 mT magnetic field) at 17 sites on each lower limb (dosage: 30 J per site). The data were analysed by two-way repeated measures analysis of variance (ANOVA, time vs experimental group) with post-hoc Bonferroni correction.

**Results:**

The percentage of change in time until exhaustion and in maximum oxygen consumption was higher in the PBMT/sMF + PBMT/sMF group than in the Placebo+Placebo group at all time-points (*p* < 0.05). Moreover, the percentage of decrease in body fat at the 16th week was higher in the PBMT/sMF + PBMT/sMF group than in the Placebo+Placebo group (*p* < 0.05).

**Conclusions:**

PBMT/sMF can potentiate the effects of aerobic endurance training and decrease performance loss after a 4-week detraining period. Thus, it may prove to be an important tool for both amateur and high-performance athletes as well as people undergoing rehabilitation.

**Trial registration:**

NCT03879226. Trial registered on 18 March 2019.

## Background

Regular physical activity is recommended for improving general health, performance enhancement, and the rehabilitation of chronic diseases [1, 2]. In addition, aerobic exercise improves cardiovascular health and decreases body fat mass [3–7]. However, individuals often discontinue exercise in response to illness, injury, or other factors that may alter their capacity for physical activity, causing a rapid loss of aerobic conditioning that can be observed after 2–4 weeks [8, 9]. Currently, there is insufficient evidence to support the ability of any particular resource or method to decrease the effects of detraining.

Photobiomodulation therapy (PBMT) is a therapy that uses non-ionising light sources, such as lasers, light-emitting diodes (LEDs), and broadband light, from the visible to the infrared spectrum [10]. PBMT elicits a nonthermal process where light interacts with chromophores leading to photophysical and photochemical reactions in different tissues, thereby promoting cell metabolism modulation [11, 12]. PBMT has been investigated for promoting exercise-related ergogenic effects [10]. Leal-Junior et al. (2009), Antonialli et al. (2014), and de Paiva et al. (2016) observed that the use of PBMT with exercise can reduce creatine kinase (CK) activity through its protective effects on skeletal muscle tissue, allowing for a faster recovery [13–15]. Other studies indicate that PBMT can decrease blood lactate levels and thus, help to improve performance during exercise [16–18].

Static magnetic fields (sMF) are force fields that are produced by moving electrical currents that act on other mobile charges, which can interact with several biological processes [19, 20], also leading to the modulation of cellular metabolism [20–22]. Previous studies have reported that the use of sMF in association with PBMT generates greater effects in cellular metabolism than the use of PBMT alone [23]. In the clinical context, it has been demonstrated that the association of PBMT with sMF (PBMT/sMF) promotes ergogenic effects [18, 24]. Moreover, Miranda et al. (2018) verified that this combination enhances the performance of individuals during an aerobic training program, increasing the percentage of change of maximum oxygen consumption (VO_2_max) and time until exhaustion after 12 weeks of the training protocol [25].

To the best of our knowledge, no previous study has investigated the effects of PBMT/sMF in the detraining or deconditioning period. However, the effects of PBMT on the oxidative metabolism of peripheral blood cells (erythrocytes, granulocytes, and lymphocytes), leading to an enhanced oxygen-carrying ability of blood, was previously demonstrated in heparinised blood samples [26]. According to Wasik et al., the partial pressure of oxygen (PO_2_) and oxygen saturation (SaO_2_) increases after PBMT irradiation [26]. These findings suggest that PBMT could attenuate the loss of performance observed during the detraining period after an aerobic training program through increased oxygen transportation, and consequently, the ability of muscles to use it, which is decreased during the detraining period.

Therefore, PBMT/sMF may prove to be an important tool for both amateur and high-performance athletes as well as for people in the process of rehabilitation who discontinue exercise due to illness, injury, or other factors. With this perspective, we aimed to investigate the effects of PBMT/sMF during the training and detraining period in maintaining the benefits acquired in an aerobic training program.

## Methods

The protocol and methods used in this study were previously published in a peer-reviewed scientific journal [27].

### Design and ethical aspects

A randomised, triple-blind (volunteers, therapists, and assessors), placebo-controlled clinical trial was performed at the Laboratory of Phototherapy and Innovative Technologies in Health (LaPIT). The study followed the ethical guidelines of and was approved by the Research Ethics Committee of Nove de Julho University (protocol number 1781602). The protocol was prospectively registered at ClinicalTrials.org (NCT03879226) by 18 March 2019. The first volunteer was enrolled at 25 March 2019, and all volunteers signed an informed consent form at the time of enrolment in the study.

### Subjects and sample size

As no previous studies have assessed the effects of PBMT/sMF during the detraining period after an aerobic training program, the number of participants per group in the present study was calculated based on a pilot study, with five volunteers per group, conducted by our research group to estimate the sample size. A beta value of 20% and alpha value of 5% were used to calculate the sample size.

The pilot study showed that applying PBMT/sMF during the detraining period resulted in a time to exhaustion (the primary outcome of this study) of 923.60 s (standard deviation, 65.77 s) during the progressive treadmill test; whereas applying the placebo during the detraining period resulted in a time to exhaustion of 846.82 s (standard deviation, 99.23 s). We used the Researcher’s Toolkit to calculate the sample (https://www.dssresearch.com/KnowledgeCenter/toolkitcalculators/samplesizecalculators.aspx).

Based on the parameters used to calculate the sample, we determined that 15 volunteers per group, for a total of 60 volunteers, was appropriate. Therefore, predicting a 20% sample loss, up to 72 healthy and physically inactive male volunteers aged 18–35 years who were students at the Nove de Julho University, would be recruited for the study to ensure a final sample size of 60 volunteers. As the PBMT/sMF device used in the study has no harmful thermal effects, volunteers of all ethnicities were recruited [28].

### Patient and public involvement statement

Patients and/or the public were not involved in the design, recruitment to, and conduct of this study. At the end of the study, the main results were disseminated to participants by email.

### Eligibility criteria

#### Inclusion criteria

Healthy men aged 18–35 years from all ethnicities, who were non-smokers, with no history of a musculoskeletal injury in the hip and knee regions in the 2 months before the study, who did not regularly use pharmacological agents and/or nutritional supplements, and who completed at least 80% of the study procedures, were included in the study.

#### Exclusion criteria

Volunteers who showed any musculoskeletal injury in the 2 months before the study, were injured during the study, regularly used any type of nutritional supplement or pharmacological agent, or who showed signs and symptoms of any neurological, metabolic, inflammatory, pulmonary, oncological, or cardiovascular disease that may limit the execution of high-intensity exercises, were excluded from the study.

### Randomisation and blinding and experimental groups

In order to avoid selection bias, and to ensure that all individuals were randomly allocated to any group, balanced block randomisation was performed based on the primary outcome (time to exhaustion in the progressive treadmill test) by a researcher who had no contact with the study subjects or the other researchers involved in the project.

A researcher programmed the device (PBMT/sMF or placebo) and was instructed not to inform the volunteers or other researchers of the type of treatment (PBMT/sMF or placebo). The sounds and signals emitted from the device as well as the information displayed on the screen were identical, regardless of the type of treatment (PBMT/sMF or placebo), providing the appropriate blinding of volunteers and therapists. All volunteers used opaque glasses during the treatments both for safety and to aid in blinding. Thus, volunteers, evaluators, and therapists were blinded to maintain the triple-blind design.

Randomisation labels were created through the random.org website, and a series of sealed, opaque, and numbered envelopes were used to ensure confidentiality and to determine to which experimental group each volunteer was to be allocated. Volunteers were allocated as described below:

PBMT/sMF + PBMT/sMF: PBMT/sMF before and after the aerobic training sessions (12 weeks, 3 times a week) and PBMT/sMF during the detraining period (4 weeks, 3 times a week).

PBMT/sMF + Placebo: PBMT/sMF before and after the aerobic training sessions (12 weeks, 3 times a week) and placebo during the detraining period (4 weeks, 3 times a week).

Placebo+PBMT/sMF: Placebo before and after the aerobic training sessions (12 weeks, 3 times a week) and PBMT/sMF during the detraining period (4 weeks, 3 times a week).

Placebo+Placebo: Placebo before and after the aerobic training sessions (12 weeks, 3 times a week) and placebo during the detraining period (4 weeks, 3 times a week).

The individuals were subjected to 12 consecutive weeks of aerobic endurance training on a motorised treadmill, with 3 training sessions per week on non-consecutive days.

After the 12-week training period, the volunteers received the treatment (either PBMT/sMF or placebo, depending on the group to which they were allocated) during the 4 weeks (3 times a week) without training.

**Fig. 1:**
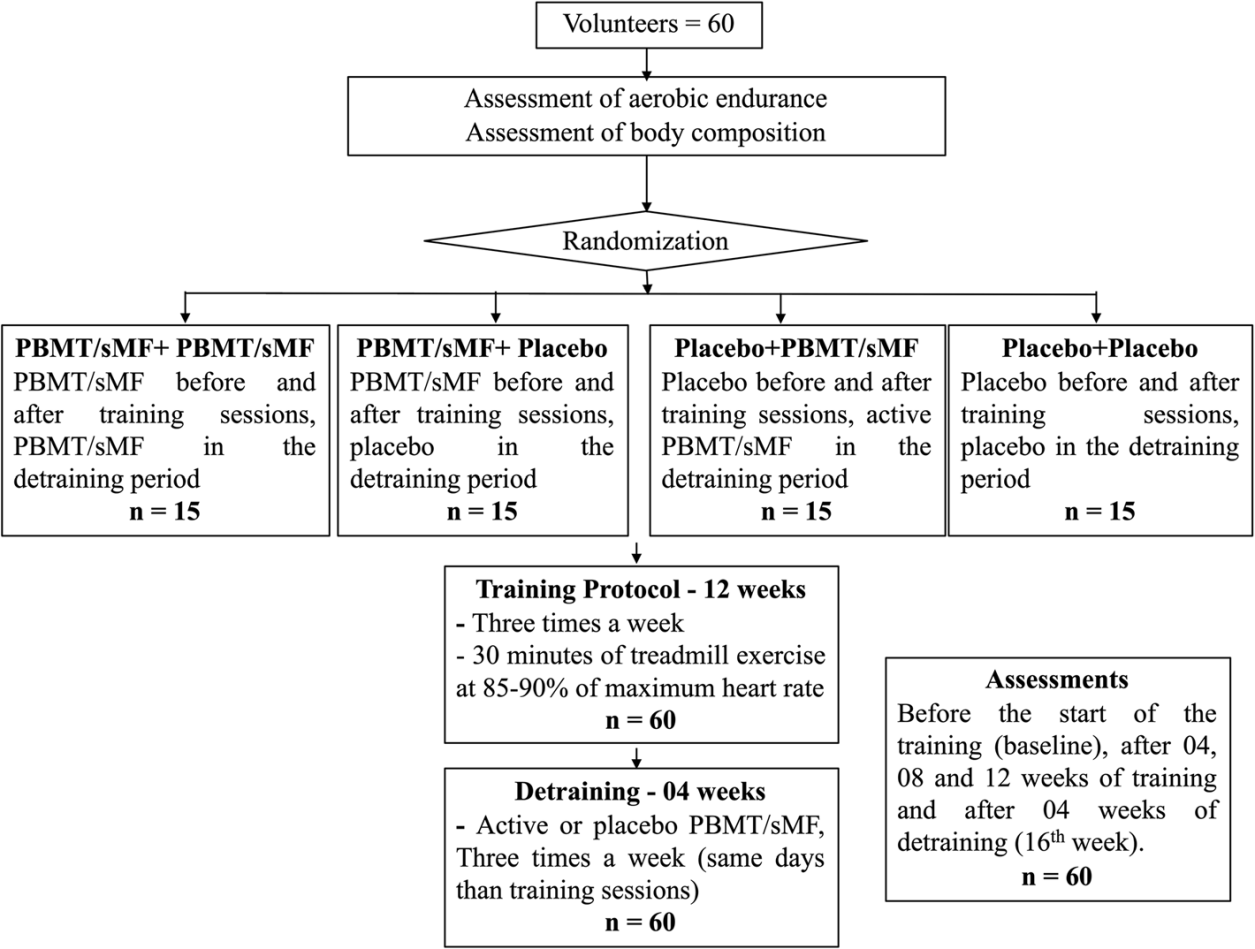
CONSORT flowchart

The evaluations described below were performed before starting the protocol (baseline) and after 4, 8, and 12 weeks of aerobic endurance training as well as after 4 weeks without training (detraining period) at the 16th week. A flowchart summarising the procedures of this study is presented in Fig. 1.

### Procedures

#### Cardiorespiratory evaluation

Ergospirometry is currently the most accurate cardiorespiratory fitness test [29]. In this study, we used a progressive treadmill protocol previously used by our research group [25, 30–32]. An ergometric treadmill model Super ATL AQ5, and a gas analyser model VO2000, which was connected to a microcomputer for data visualisation and recording were used. The ergospirometry test started with the treadmill set at a 1% slope, with an initial speed of 3 km/h, which was maintained until the end of the 3-min warm-up phase. After the warm-up phase, the speed was increased by 1 km/h every minute until a maximum speed of 16 km/h was reached. The end of the test was defined by the volunteers, who were instructed to perform the test until they reached exhaustion [25, 30–32]. The recovery phase then began, lasting 3 min at a speed of 6 km/h. During the test, data on the total time of the exercise (time to exhaustion), maximum oxygen uptake in absolute and relative values in relation to body mass (VO_2max_) were recorded [25, 30–32]. The chosen parameters represent those that are most commonly used for this purpose, and as such, these data were used to assess the performance of the subjects in the exercise protocol [29].

The entire test was monitored by electrocardiography and blood pressure measurements. If any abnormal changes in heart rate or blood pressure were found, or if the volunteer had any complaint, the test was discontinued, and the volunteer was excluded from the study.

#### Body composition evaluation

All body composition evaluations were performed by the same technician (level II of the International Society for the Advancement of Kineanthropometry - ISAK), using the procedures established by the ISAK [33]. The height, body mass, length of body segments, diameters, perimeters, and skinfolds of the participants were measured to assess the muscle mass, adipose mass, residual mass, bone mass, and epithelial mass [25].

#### Aerobic training

Aerobic training was performed on a treadmill, with and without PBMT/sMF, 3 times a week on non-consecutive days, for 12 weeks. Each training session was supervised by a certified trainer and lasted 30 min. The speed of the motorised treadmill was tailored to each participant for every training session. The speed of the treadmill was varied during the exercise to keep the heart rate of the volunteers between 85 to 90% of maximum heart rate [25]; the heart rate of the volunteers was monitored during the entire exercise session using a heart rate monitor. The maximum heart rate was determined during the cardiorespiratory evaluation protocol described above [25, 31].

The protocol was interrupted when criteria established by the American Heart Association guidelines were met. The subjects were also evaluated using the 0–10 Borg scale, which is a simple method for classifying perceived exertion, feelings of physical fatigue, or dyspnoea.

#### Photobiomodulation therapy (PBMT) and static magnetic fields (sMF)

**Fig. 2:**
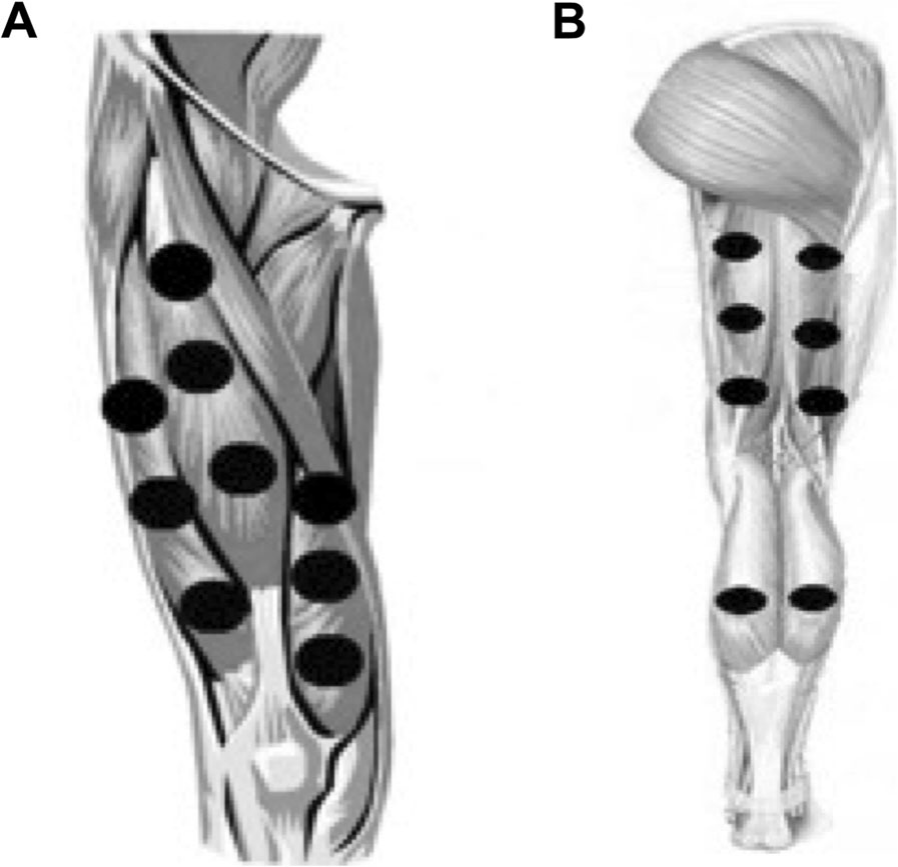
**a**: Treatment sites at knee extensor muscles **b**: Treatment sites at knee-flexor and ankle plantar-flexor muscles

PBMT/sMF or placebo were applied before and after each training session, as aforementioned. This irradiation protocol was tested and optimised in a previous study conducted by our research group [25]. The results of this study showed that PBMT/sMF before and after each training session was the most effective in enhancing the effects of aerobic training [25]. PBMT/sMF was applied bilaterally using the direct contact method with light pressure on the skin at different sites, namely nine sites on the knee extensor muscles (Fig. 2a), six sites on the knee flexor muscles, and two sites on the plantar flexor muscles (Fig. 2b).

To apply PBMT/sMF, a 12-diode cluster was used, including four 905 nm laser diodes (12.5 W peak power of each diode, 250 Hz), four 875 nm LEDs (average power of each diode, 17.5 mW), four 640 nm LEDs (average power of each diode, 15 mW), and a static magnetic field (35 mT) manufactured by Multi Radiance Medical (Solon, OH - USA). Considering the large irradiation area used in the present project, the use of diode clusters was essential for the application of PBMT/sMF; the cluster was circular and had a total irradiation area of 20 cm^2^.

The dose for active PBMT/sMF was 30 J per area (228 s of irradiation in each area) [14], 510 J of irradiated energy per lower limb [18, 25, 31], and 1020 J of total irradiated energy [18, 25, 31]. The dose used at each site was previously tested and optimised by our research group using the same PBMT/sMF device and demonstrated favourable results in terms of enhancing performance and muscle recovery [14]. Furthermore, the irradiation sites were also previously optimised by our research group [18, 25, 31]. The complete description of PBMT/sMF parameters is presented in Table 1.

**Table 1:** Parameters for PBMT/sMF

Number of lasers	4 Super-pulsed (infrared)
Wavelength (nm)	905 (±1)
Frequency (Hz)	250
Peak power (W) - each	12.5
Average mean optical output (mW) – each	0.3125
Power density (mW/cm^2^) - each	0.71
Energy density (J/cm^2^) - each	0.162
Dose (J) - each	0.07125
Spot size of laser (cm^2^) - each	0.44
Number of red LEDs	4 Red
Wavelength of red LEDs (nm)	640 (±10)
Frequency (Hz)	2
Average optical output (mW) - each	15
Power density (mW/cm^2^) - each	16.66
Energy density (J/cm^2^) - each	3.8
Dose (J) - each	3.42
Spot size of red LED (cm^2^) - each	0.9
Number of infrared LEDs	4 Infrared
Wavelength of infrared LEDs (nm)	875 (±10)
Frequency (Hz)	16
Average optical output (mW) - each	17.5
Power density (mW/cm^2^) - each	19.44
Energy density (J/cm^2^) - each	4.43
Dose (J) - each	3.99
Spot size of LED (cm^2^) - each	0.9
Magnetic Field (mT)	35
Irradiation time per site (sec)	228
Total dose per site (J)	30
Total dose applied per lower limb (J)	510
Aperture of device (cm^2^)	20
Application mode	Cluster probe held stationary in skin contact with a 90-degree angle and slight pressure

### Statistical analysis

The primary outcome of this study was time until exhaustion, obtained from the progressive treadmill test. The secondary outcomes were VO_2_max in relation to body mass and body fat percentage. The intention-to-treat analysis was followed a priori, and all data were analysed by a blinded researcher who was not involved in the data collection. The findings were tested for normality using the Shapiro–Wilk test and were determined to have a normal distribution. Data were expressed as the mean and standard deviation and were analysed by two-way repeated measures analysis of variance (ANOVA, time vs experimental group) with post-hoc Bonferroni correction. Data were also analysed in terms of the absolute values and the percentage of change based on the values established at baseline. The significance level was set at *p* < 0.05. In the graphs, data are expressed as the mean and standard error of the mean (SEM).

## Results

All 60 participants completed the full 16-week study; there were no dropouts and there were no adverse effects reported. The characteristics of the volunteers are summarised in Table 2. Statistical analysis revealed that there were no significant differences (*p* > 0.05) between the volunteers from the four experimental groups with respect to the participants’ characteristics.

**Table 2:** Participants’ characteristics in absolute values

	PBMT/sMF + PBMT/sMF	PBMT/sMF + Placebo	Placebo + PBMT/sMF	Placebo + Placebo
Age (years)	24.79 ± 5.22	23.82 ± 4.29	23.81 ± 6.01	28.83 ± 5.52
Body mass (kg)	78.09 ± 16.49	73.71 ± 14.33	71.24 ± 13.50	79.99 ± 12.12
Height (cm)	174.71 ± 7.32	175.94 ± 4.94	173.88 ± 6.67	173.17 ± 7.59
Body mass index^a^	25.55 ± 5.16	23.78 ± 4.43	23.52 ± 4.10	26.62 ± 3.17
Heart rate (beats per minute)	82.21 ± 11.34	80.65 ± 13.93	84.13 ± 14.12	84.08 ± 11.14
Systolic blood pressure (mmHg)	114.29 ± 5.56	117.07 ± 14.04	110.00 ± 8.94	120.00 ± 18.59
Diastolic blood pressure (mmHg)	80.00 ± 5.55	81.18 ± 7.81	77.50 ± 7.75	85.00 ± 13.82

^a^ Calculated as kg/m^2^; VO2 max: oxygen uptake. Data is expressed in average and standard deviation (±)

Table 3 shows the results of the progressive cardiopulmonary test in absolute values for the different variables analysed in all experimental groups of this study. There were no statistically significant differences in time until exhaustion or body fat percentage between the groups. Regarding the VO_2_max, all groups treated with PBMT/sMF showed an increase in VO_2_max over time when compared to the Placebo+Placebo group. This difference was statistically significant in the PBMT/sMF + PBMT/sMF group (*p* < 0.05, *p* < 0.05, *p* < 0.05, and *p* < 0.01) and the PBMT/sMF + Placebo group (*p* < 0.01, *p* < 0.001, *p* < 0.001, and *p* < 0.05) at all experimental time-points (4th, 8th, 12th, and 16th weeks, respectively).

**Table 3:** Progressive endurance test variables in absolute values

	Baseline	4 weeks	8 weeks	12 weeks	16 weeks
**Time until exhaustion (sec)**					
**PBMT/sMF + PBMT/sMF**	767.29 ± 77.58	887.86 ± 81.37	921.21 ± 101.07	986.86 ± 125.53	932.57 ± 110.46
**PBMT/sMF + Placebo**	812.53 ± 121.46	934.35 ± 134.16	980.24 ± 149.28	1025.65 ± 165.58	915.53 ± 147.63
**Placebo + PBMT/sMF**	853.54 ± 146.12	906.5 ± 167.54	949.25 ± 176.76	961 ± 153.46	940.06 ± 142.37
**Placebo + Placebo**	801.67 ± 122.49	855.83 ± 134.66	853 ± 119.39	892.67 ± 155.03	823.08 ± 160.85
**VO**_**2 max**_ **(mL/kg/min)**					
**PBMT/sMF + PBMT/sMF**	21.94 ± 2.71	30.81 ± 4.38^*^	30.87 ± 4.49^*^	30.64 ± 4.98^*^	30.26 ± 5.42^**^
**PBMT/sMF + Placebo**	24.91 ± 6.08	32.06 ± 8.53^**^	33.48 ± 7.97^***^	33.34 ± 9.13^***^	29.46 ± 8.16^*^
**Placebo + PBMT/sMF**	24.61 ± 6.14	26.93 ± 7.25	28.27 ± 6.41	27.4 ± 5.51	28.15 ± 5.44
**Placebo + Placebo**	22.06 ± 5.12	24.68 ± 4.96	24.89 ± 5.60	24.34 ± 5.24	22.38 ± 5.31
**Fat percentage**					
**PBMT/sMF + PBMT/sMF**	24.55 ± 10.64	22.61 ± 9.07	22.3 ± 8.74	21.59 ± 9.58	20.84 ± 8.65
**PBMT/sMF + Placebo**	26.16 ± 6.46	25.39 ± 7.31	25.19 ± 6.7	24.85 ± 7.21	25.2 ± 7.98
**Placebo + PBMT/sMF**	18.8 ± 10.39	17.83 ± 10.00	17.62 ± 10.00	16.96 ± 9.14	17.24 ± 9.19
**Placebo + Placebo**	21.05 ± 9.60	20.29 ± 9.24	19.91 ± 8.89	19.43 ± 8.56	19.13 ± 8.53

Data are expressed as means and standard deviations (±). VO_2_ max: maximum oxygen uptake. ^*^ indicates a statistically significant difference compared to Placebo+Placebo (*p* < 0.05), ^**^ indicates a statistically significant difference compared to Placebo+Placebo (*p* < 0.01), ^***^ indicates a statistically significant difference compared to Placebo+Placebo (*p* < 0.001)

**Fig. 3:**
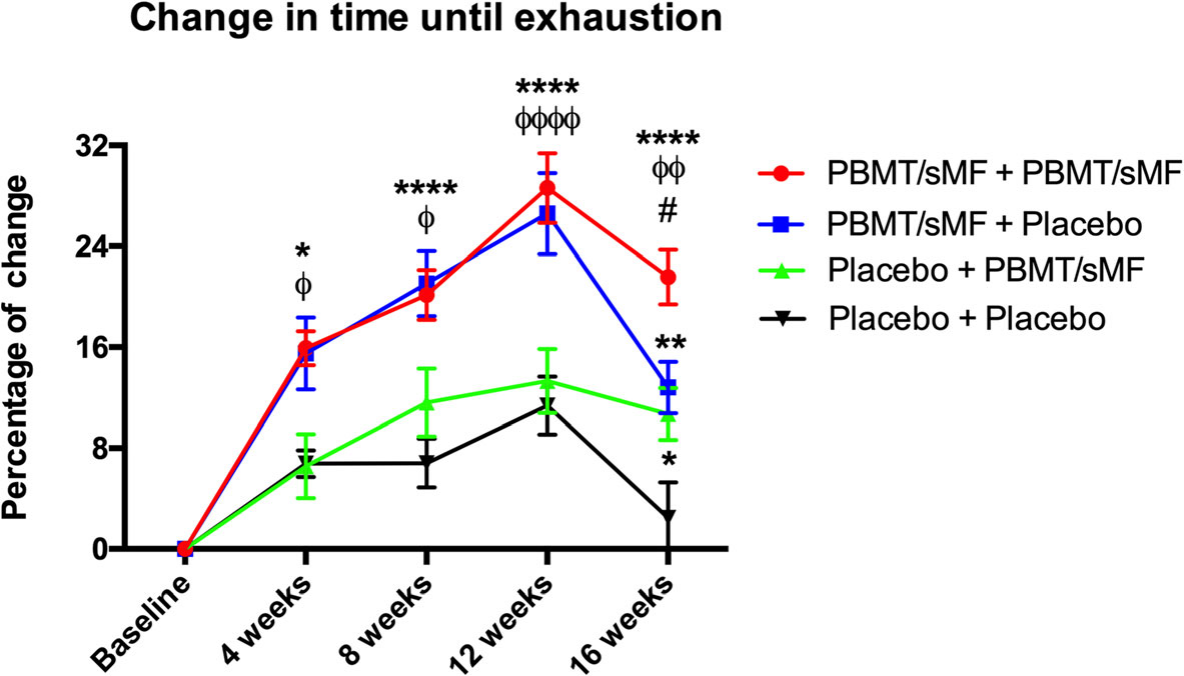
Percentage of change in time to exhaustion. The data are presented in mean and SEM. * indicates statistical significance of *p* < 0.05 compared to Placebo+Placebo; ** indicates statistical significance of *p* < 0.01 compared to Placebo+Placebo; **** indicates statistical significance of *p* < 0.0001 compared to Placebo+Placebo; ^ø^ indicates statistical significance of *p* < 0.05 compared to Placebo+PBMT/sMF; ^øø^ indicates statistical significance of *p* < 0.01 compared to Placebo+PBMT/sMF; ^øøøø^ indicates statistical significance of *p* < 0.0001 compared to Placebo+PBMT/sMF; and ^#^ indicates statistical significance of *p* < 0.05 compared to PBMT/sMF + Placebo

With respect to the time until exhaustion, Fig. 3 shows the percentage of change in the evaluated time-points. The PBMT/sMF + PBMT/sMF group showed a statistically significant difference (*p* < 0.05, *p* < 0.0001, *p* < 0.0001, and *p* < 0.0001) at all experimental time-points (4th, 8th, 12th, and 16th week, respectively) when compared to the Placebo+Placebo group. In the detraining period (16th week), the PBMT/sMF + PBMT/sMF group also showed a statistically significant difference (*p* < 0.05) compared to the PBMT/sMF + Placebo group. Moreover, the group treated with PBMT/sMF only in the training period (PBMT/sMF + Placebo) or in the detraining period (Placebo+PBMT/sMF), showed a statistically significant difference (*p* < 0.01 and *p* < 0.05, respectively) in the percentage of change of time until exhaustion when compared to the placebo.

**Fig. 4:**
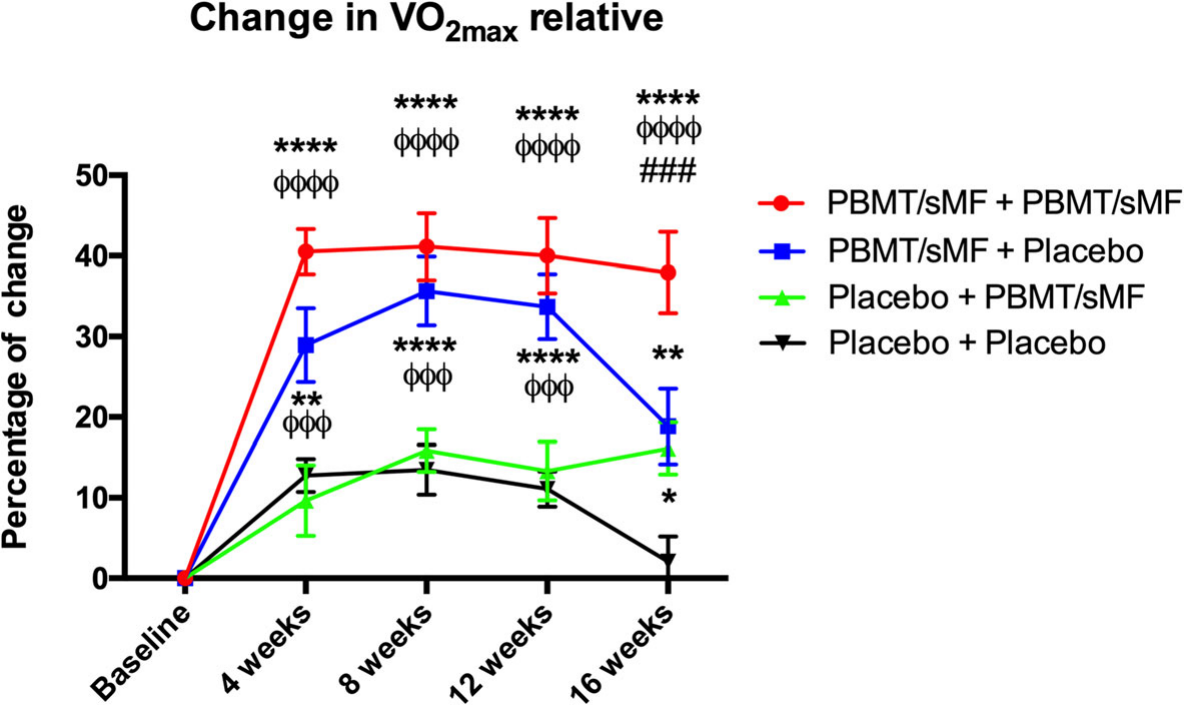
Percentage of change in relative maximum oxygen uptake. The data are presented in mean and SEM. * indicates statistical significance of *p* < 0.05 compared to Placebo+Placebo; ** indicates statistical significance of *p* < 0.01 compared to Placebo+Placebo; **** indicates statistical significance of *p* < 0.0001 compared to Placebo+Placebo; ^øøø^ indicates statistical significance of *p* < 0.001 compared to Placebo+PBMT/sMF; ^øøøø^ indicates statistical significance of *p* < 0.0001 compared to Placebo+PBMT/sMF; ^###^ indicates statistical significance of *p* < 0.001 compared to PBMT/sMF + Placebo

Figure 4 graphically represents the percentage of change in VO_2_max in relation to body mass in the study participants. The PBMT/sMF + PBMT/sMF group showed a statistically significant difference (*p* < 0.0001, *p* < 0.0001, *p* < 0.0001, and *p* < 0.0001) when compared to the Placebo +Placebo group, at all experimental time-points (4th, 8th, 12th, and 16th week, respectively). In the 16th week, the PBMT/sMF + PBMT/sMF group also presented a statistically significant difference compared to the Placebo+PBMT/sMF group (*p* < 0.0001) and the PBMT/sMF + Placebo group (*p* < 0.001). The groups that received PBMT/sMF only in the training period (PBMT/sMF + Placebo) or in the detraining period (Placebo+PBMT/sMF), also presented a statistically significant difference (*p* < 0.01 and *p* < 0.05, respectively) compared to the Placebo+Placebo group at the 16th week.

**Fig. 5:**
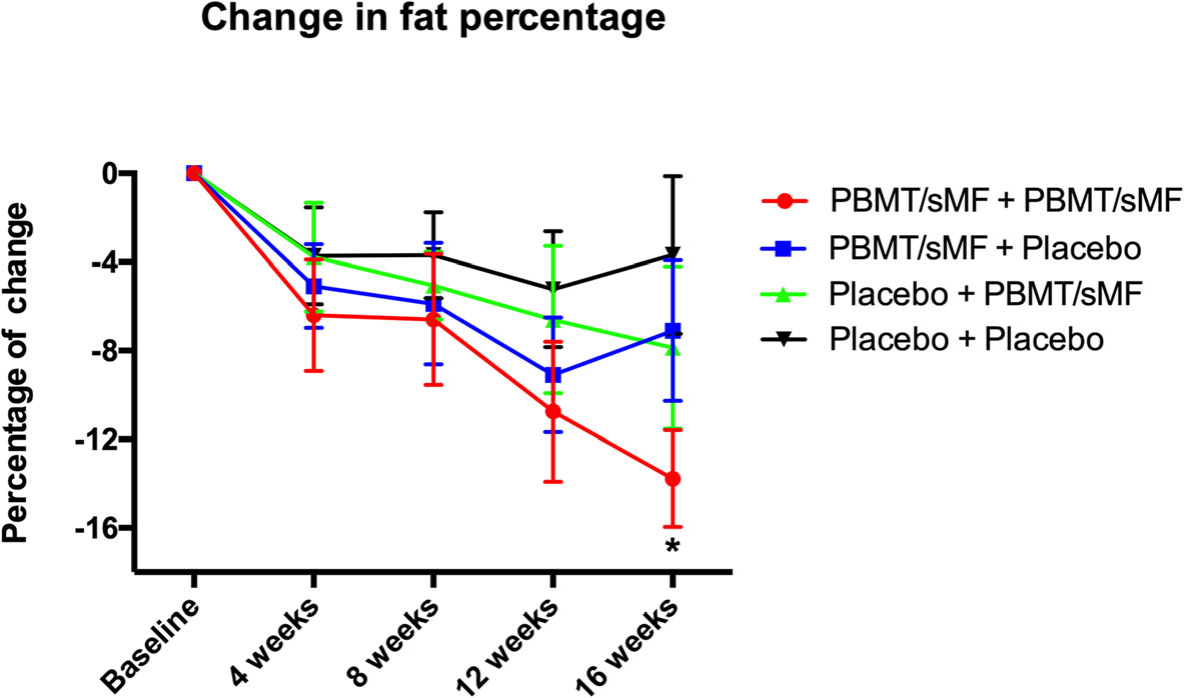
Percentage of change in body fat. The data are presented in mean and SEM. * indicates statistical significance of *p* < 0.05 compared to Placebo+Placebo

Figure 5 shows the percentage of change in body fat percentage throughout the study. Only the group treated with PBMT/sMF throughout the whole study (PBMT/sMF + PBMT/sMF) demonstrated a percentage of change statistically superior (*p* < 0.05) in body fat mass compared to the Placebo+Placebo group, at the 16th week. There were no significant differences in the other experimental groups or time-points tested.

## Discussion

It is important to highlight that this was the first study to evaluate the effects of PBMT and sMF (PBMT/sMF) in the detraining period after aerobic training. In the present study, the application of PBMT/sMF before and after each training session, 3 times a week for 12 weeks, led to a statistically significant increase in the absolute values of VO_2_max at all evaluated time-points for the PBMT/sMF + PBMT/sMF group and the PBMT/sMF + Placebo group compared to the Placebo+Placebo group. These positive results corroborate previous findings by our research group [25] as well as those reported by Paolillo et al. [34, 35]. Moreover, we observed that in the final training phase (12th week), PBMT/sMF improved the percentage of change of VO_2_max in 40.02% and time until exhaustion in 28.62%. Again, our results further validate the findings of Miranda et al. [25] and Paolillo et al. [25, 34, 35], thereby increasing the body of evidence supporting the benefits of PBMT in association with aerobic training.

As previously mentioned, the main goal of this study was to investigate the effects of PBMT/sMF during a 4-week period without physical activity. At the end of this period, the group that received PBMT/sMF throughout the whole study (PBMT/sMF + PBMT/sMF group) showed a statistically significant difference in the absolute values (*p* < 0.01) of VO_2_max compared to the Placebo+Placebo group. Interestingly, PBMT/sMF applied only during the training phase (PBMT/sMF + Placebo) also led to a statistically significant increase (*p* < 0.05) in the absolute values of VO_2_max compared to the absolute values observed in the Placebo+Placebo group in the 16th week.

Previous studies have shown that a period of physical inactivity, specifically between 2 to 4 weeks [8], can lead to significant losses in aerobic capacity [5, 8]. In our study, we also observed a decrease in percentage of change of VO_2_max in the Placebo+Placebo and PBMT/sMF + Placebo groups during the detraining period (16th week) compared to the training period (12th week). Despite the lack of consensus regarding the magnitude of the loss of aerobic capacity provoked by a period of no physical activity, we believe that decreases in percentage of change of VO_2_max observed in the Placebo+Placebo and PBMT/sMF + Placebo groups are mainly related to the initial level of physical fitness of the practitioners as well as the duration of the detraining period, as described in the literature [8].

Similarly, the percentage of change in time until exhaustion was significantly higher in the PBMT/sMF + PBMT/sMF group than in the Placebo+Placebo group (21.55% versus 2.46%) in the 16th week. Moreover, the Placebo+PBMT/sMF group showed better results than the Placebo+Placebo group (10.7% versus 2.46%) in the 16th week. The group that received PBMT/sMF throughout the entire training and detraining periods also showed an increased change in percentage of fat. However, this same group showed a statistically significant decrease in this percentage, in relation to the Placebo+Placebo group, only in the detraining period (16th week). These findings allow us to infer that the application of PBMT/sMF throughout the whole study aided in reducing the body fat of the participants, even during a period without training. Similar findings were previously presented with the use of PBMT/sMF applied before and after aerobic training sessions [25]. These findings reiterate the increased effectiveness of PBMT/sMF when applied in both training and detraining periods.

The positive results obtained from the use of PBMT/sMF can be attributed to its ergogenic effects on aerobic training, similar to the findings of Miranda et al. [25]. Moreover, the parameters used for irradiation in the present study are in line with those recently recommended by Leal-Junior et al. [10]. The establishment of optimised parameters is paramount for the effectiveness of PBMT/sMF since, in addition to the dose, the moment to perform the treatment and application sites should also be considered for therapy optimisation [36]. Given the importance of these factors, the protocol for irradiation followed the same parameters as those previously tested by Miranda et al. [25], which showed positive effects for the percentage of change in VO_2_max, time until exhaustion, and body fat.

We believe that our findings are of great importance, especially in both rehabilitation and sports scenarios. It is well-known how difficult it is for elite athletes to maintain their performance levels, especially due to frequent injuries and consequent removal from their training routine. In this regard, our findings suggest that PBMT/sMF can be an efficient alternative to physical trainers, athletes, and coaches in periods where aerobic training is interrupted.

Our findings may also bring up the question of whether PBMT/sMF is an acceptable technique for training and detraining in competitive and professional sports. The current version of the World Anti-Doping Code, published by the World Anti-Doping Agency (WADA) in 2015 [37], states that a substance or method can be considered as doping if two of the following three criteria are fulfilled: ‘1 - Medical or other scientific evidence, pharmacological effect or experience that the substance or method, alone or in combination with other substances or methods, has the potential to enhance or enhances sport performance; 2 - Medical or other scientific evidence, pharmacological effect or experience that the use of the substance or method represents an actual or potential health risk to the athlete; and 3 - WADA’s determination that the use of the substance or method violates the spirit of sport described in the introduction to the code’. Furthermore, WADA also states that ‘A substance or method shall also be included on the Prohibited List if WADA determines there is medical or other scientific evidence, pharmacological effect or experience that the substance or method has the potential to mask the Use of other Prohibited Substances or Prohibited Methods’.

However, since, as far as we know, PBMT/sMF does not have side effects or potential health risks, it is likely not fulfilling criterion number 2. Moreover, as the last criterion is more political than scientific, the decision about whether PBMT/sMF use should be included in the prohibited list of WADA is for the members of the executive board committee to decide.

A limitation of our study is that we did not investigate the mechanisms of action behind the positive effects presented by PBMT/sMF, which limits us to provide mechanistic insights related to the ergogenic effects of the therapy. Despite the wide number of reports regarding the modulatory effects of PBMT/sMF in cellular metabolism [11, 12, 23] and on the partial pressure of oxygen and oxygen saturation in peripheral blood cells [26], we believe that further research should focus on the mechanisms of action behind the ergogenic effects of PBMT/sMF in humans. A second limitation of this study is that we did not monitor the habitual activity of our participants during detraining, which should also be considered in further research. The last limitation of this study is that only 4 weeks of detraining after 12 weeks of aerobic endurance training were analysed; however, the attenuation of the losses due to the interruption of physical activity is of great value, both for professional and amateur athletes. These benefits can also be transferred to several different scenarios, such as individuals affected by illness and those who are bedridden or unable to perform physical exercises for rehabilitation.

## Conclusions

In the present study, the use of PBMT/sMF was able to potentiate the effects of aerobic training and reduce losses caused by the detraining period. Therefore, we believe that the results of the study are relevant and suggest a new perspective on the use of PBMT/sMF by recognising its effectiveness in maintaining the benefits obtained after aerobic training, during the detraining period.

## Data Availability

The datasets used and/or analysed during the current study are available from the corresponding author on reasonable request. Correspondence to: Ernesto Cesar Pinto Leal-Junior, Prof. Ph.D., PT. Laboratory of Phototherapy and Innovative Technologies in Health (LaPIT). Rua Vergueiro, 235/249, ZIP Code: 01504–001. São Paulo - SP, Brazil. Tel: + 55 11 3385–9134. e-mail: ernesto.leal.junior@gmail.com

## Abbreviations

PBMT: Photobiomodulation therapy
LEDs: Light-emitting diodes
sMF: Static magnetic field
VO_2_max: Maximum oxygen consumption
CK: Creatine kinase
PO_2_: Partial pressure of oxygen
SaO_2_: Oxygen saturation
